# Outdoor education on the mental health of Chinese children: a mini-review

**DOI:** 10.3389/fpsyg.2026.1881351

**Published:** 2026-10-12

**Authors:** Xin Li, Liping Zhong

**Affiliations:** Hunan University of Technology, Zhuzhou, China

**Keywords:** children, China, mental health, outdoor activity, outdoor education

## Abstract

Children's mental health is an increasing concern in education and public health. Outdoor education and related outdoor activities have therefore received growing attention. However, definitions of outdoor education remain inconsistent, and evidence concerning Chinese children is fragmented. This review addresses two questions: What is outdoor education, and what potential implications may it have for the mental health of children in China? Drawing on the PRISMA reporting framework, we searched Web of Science and Scopus, supplemented by Google Scholar, and included 18 English-language empirical studies. Sources were synthesized narratively: conceptual sources clarified the definition of outdoor education, whereas empirical studies were classified as direct or closely related intervention evidence or as indirect evidence concerning related outdoor and nature-based activities. Outdoor education is understood as structured, goal-oriented, experience-based learning in outdoor or natural settings, often aligned with educational objectives. Few studies in the retrieved English-language literature directly evaluated structured outdoor education programmes among Chinese children. This apparent scarcity may partly reflect the English-language restriction and the omission of Chinese-language databases, rather than the full extent of research in China. Studies of related activities, including nature contact, horticultural activity, and outdoor physical activity, were considered as indirect or adjacent evidence. Overall, the available evidence suggests that structured outdoor and nature-based educational activities may be associated with favorable emotional, social, and self-regulatory outcomes. By distinguishing direct from indirect evidence, this review clarifies the current evidence base and provides a foundation for contextually appropriate program development and future research in Chinese children.

## 1 Introduction

Over the past two decades, research on outdoor education has expanded, alongside its application in schools and mental health care. Studies have examined its theoretical foundations and pedagogical roles in school settings (Becker et al., 2017; Mann et al., 2022). Research has also explored potential benefits for children's psychological wellbeing, including better emotional regulation, reduced stress, greater happiness, and a stronger sense of self (Mygind et al., 2018; Rickinson et al., 2004; Samsudin et al., 2019; Tang et al., 2015). These findings have encouraged interest in the relevance of outdoor education and related experiences to children's mental health.

Despite this interest, definitions of outdoor education remain inconsistent. Priest (1986) described it as an experiential learning approach involving multiple senses and cross-curricular activities that explore relationships between people and nature. Others describe regular subject instruction using local environments outside school (Barfod et al., 2021; Bentsen et al., 2009). Although these definitions differ, they share an emphasis on experience and educational purposes. This review defines outdoor education as structured, goal-oriented experiential learning in outdoor, natural, or real-world settings, typically supported by educators or programs. By contrast, outdoor activity includes time outdoors, outdoor physical activity, and nature-based experiences without necessarily involving explicit educational design. This distinction helps clarify which experiences qualify as outdoor education and which provide only related evidence.

Mental health likewise extends beyond the absence of mental illness. Galderisi et al. (2015) described a broader concept encompassing emotional regulation, emotional wellbeing, personal agency, and community support. Accordingly, examining outdoor education's potential influence on school-aged children requires consideration of multiple dimensions of psychological wellbeing rather than mental disorders alone.

Mental health concerns among Chinese children and adolescents provide an important context for this inquiry. A national survey reported that 17.5% of children aged 6–16 had a mental disorder (Li et al., 2022). Meta-analyses also indicate high detection rates of depression, anxiety, self-harm, and sleep disorders across educational stages (Rao et al., 2019). Other studies identify overlapping risks, including academic pressure, insufficient family support, being left behind, negative peer relationships, unhealthy lifestyles, and childhood adversity (Fellmeth et al., 2018; Hao et al., 2024). These problems reflect interactions among family, school, social, and individual factors, supporting further investigation of whether outdoor education could help address these challenges. Given this situation, outdoor education could be a way to address mental health challenges.

In recent years, outdoor education has received increasing attention, as reflected in its growing prominence in education policy (Ministry of Education et al., 2016). However, the reviews cited above did not specifically synthesize evidence concerning Chinese children. Their educational, social, and cultural contexts may influence both the implementation of outdoor education and its potential mental health implications. Direct evidence linking clearly defined outdoor education to mental health outcomes in this population remains limited. Much of the available literature instead examines related experiences, including nature exposure, horticultural activities, outdoor physical activity, and time outdoors.

Against this background, this review addresses two questions: What is outdoor education, and what potential impacts may it have on children's mental health in China? By distinguishing educational interventions from broader outdoor experiences, the review aims to clarify the concept and improve understanding of its potential implications for Chinese children. This distinction is particularly important when interpreting evidence from activities that share outdoor or natural settings but differ in educational objectives and support. The review therefore considers related evidence cautiously while acknowledging the limited research directly addressing clearly defined outdoor education.

## 2 Methodology

### 2.1 Data categories and origins

#### 2.1.1 Search approach

The article screening process drew on the PRISMA flow chart (Page et al., 2021). It was not designed as a systematic review, and no review protocol was registered. On February 15, 2026, we searched the Web of Science and Scopus databases for peer-reviewed literature on outdoor education and the mental health of Chinese children. Based on previously validated retrieval strategies, we combined keywords in multiple ways to conduct the search. For outdoor education research, we referenced the review by Down et al. (2025), the systematic review and meta-analysis included keywords such as *outdoor* education and outdoor adventure education. To identify key term variants, we consulted a series of review articles (Becker et al., 2017; Hu and Mou, 2025). Search terms were informed by relevant reviews and adapted to the scope of this mini-review.

We searched the heading and summary sections within the retrieval. Comprehensive specifics are presented in Table 1. Preliminary searches yielded 64 articles from the Web of Science database and 90 from Scopus, all of which were subsequently cataloged within the Zotero reference management system. A total of 154 records were identified and imported into Zotero. After duplicate removal, 52 records were excluded, leaving 102 articles for subsequent screening and eligibility assessment.

**Table 1:** Search strategies and results in the Web of Science and Scopus databases.

Step	Terms	Results
Database: web of science		
1	TS = (“Outdoor education” OR “outdoor activity” OR “outdoor play” OR “Experiential education” OR “Field trip” OR “Experiential tourism” OR “Study tourism” OR “Learning travel” OR “outdoor education” OR “adventure education” OR “outdoor adventure education” OR “adventure learning” OR “outdoor learning” OR “outdoor teaching” OR “learning outside the classroom” OR “out-of-classroom” OR “experiential learning” OR “expeditionary learning” OR “udeskole” OR “uteskole” OR “friluftsliv” OR “forest school” OR “nature school” OR “environmental education” OR “place-based education” OR “Draussenschule” OR “out-of-school physical activity” OR “outdoor physical activity” OR “outdoor activities” OR “Outdoor Activity Frequency” OR “daytime outdoor activities” OR “outdoor education intervention” OR “outdoor sport” OR “outdoor time”)	40,066
2	TS = (“Mental illness” OR “mental health” OR “Mental health” OR “Mental well” OR “Social well” OR “Emotional well” OR “Emotional well-being” OR “Psychological well” OR “Psychological Well-Being” OR “Resilience” OR “Mastery” OR “Subjective well-being” OR “Eudaimonic well-being” OR “Mental resilience” OR “Self-esteem” OR “self-esteem” OR “self-efficacy” OR “self-image” OR “self-concept” OR “positive affect” OR “optimism” OR “happiness and satisfaction with life” OR “depression” OR “anxiety” OR “stress” OR “negative affect” OR “Mental health disorders”)	4,571,278
3	TS = (“children” OR “child” OR “schoolchild” OR “adolescent” OR “teen” OR “pubescent” OR “school” OR “school-age”)	3,214,464
4	TS = (“China” OR “Chinese”)	1,814,427
5	1 AND 2 AND 3 AND 4	64
Database: scopus		
1	TITLE-ABS-KEY (“Outdoor education” OR “outdoor activity” OR “outdoor play” OR “Experiential education” OR “Field trip” OR “Experiential tourism” OR “Study tourism” OR “Learning travel” OR “outdoor education” OR “adventure education” OR “outdoor adventure education” OR “adventure learning” OR “outdoor learning” OR “outdoor teaching” OR “learning outside the classroom” OR “out-of-classroom” OR “experiential learning” OR “expeditionary learning” OR “udeskole” OR “uteskole” OR “friluftsliv” OR “forest school” OR “nature school” OR “environmental education” OR “place-based education” OR “Draussenschule” OR “out-of-school physical activity” OR “outdoor physical activity” OR “outdoor activities” OR “Outdoor Activity Frequency” OR “daytime outdoor activities” OR “outdoor education intervention” OR “outdoor sport” OR “outdoor time”)	60,094
2	TITLE-ABS-KEY (“Mental illness” OR “mental health” OR “Mental health” OR “Mental well” OR “Social well” OR “Emotional well” OR “Emotional well-being” OR “Psychological well” OR “Psychological Well-Being” OR “Resilience” OR “Mastery” OR “Subjective well-being” OR “Eudaimonic well-being” OR “Mental resilience” OR “Self-esteem” OR “self-esteem” OR “self-efficacy” OR “self-image” OR “self-concept” OR “positive affect” OR “optimism” OR “happiness and satisfaction with life” OR “depression” OR “anxiety” OR “stress” OR “negative affect” OR “Mental health disorders”)	5,980,629
3	TITLE-ABS-KEY (“children” OR “child” OR “schoolchild” OR “adolescent” OR “teen” OR “pubescent” OR “school” OR “school-age”)	6,397,810
4	TITLE-ABS-KEY (“China” OR “Chinese”)	2,385,116
5	1 AND 2 AND 3 AND 4	90

#### 2.1.2 Eligibility criteria

Two evidence sets were used. Research Question 1 drew on conceptual and contextual sources, to clarify the definition and characteristics of outdoor education. These sources were not treated as empirical evidence of mental health effects. Research Question 2 used a separate empirical evidence set. The eligibility criteria below apply only to that set, the inclusion criteria are as follows: (1) articles published in the English language; (2) articles reporting primary empirical data; (3) articles whose subjects included Chinese children participating in outdoor education or related activities; and (4) included participants primarily aged under 18 years, or reported extractable results for participants aged under 18 years. In accordance with the United Nations Convention on the Rights of the Child (UNICEF, 1989), children were defined as individuals under the age of 18. Articles were excluded if they were reviews or opinion pieces, or if they did not address outdoor education and related activities, as such studies fell outside the scope of our research questions.

Study selection was conducted independently by two reviewers, with disagreements resolved through discussion. Among the 102 screened records, three book chapters and five conference papers and eight non-English publications were excluded, leaving 86 reports for full-text retrieval. All 86 full texts were retrieved and assessed against the eligibility criteria for the empirical evidence set addressing Research Question 2. Following full-text eligibility assessment, 74 reports were excluded: 27 did not involve outdoor education or related activities, 32 did not address mental health outcomes, 10 did not concern children, four did not concern the Chinese population, and one was a systematic review. The remaining 12 studies were included in the empirical evidence set.

An additional 16 articles were identified through Google Scholar searches. Following full-text assessment, reports were included in the empirical evidence set for Research Question 2 only when they met the prespecified eligibility criteria. Four of them met the criteria. Detailed reasons are reported in the PRISMA flow diagram. Ultimately, 16 articles were included for data analysis and narrative synthesis.

#### 2.1.3 Extraction and analysis

The data extraction and analysis process drew on the work of Yang and Whatman (2025): (1) Stored the 18 manuscripts within a designated directory; (2) comprehensively reviewed these texts; (3) transferred core research variables into a multi-sheet spreadsheet matrix. Documented information encompassed researcher identities alongside publication dates, geographical context, research design, research subjects, data gathering approaches, analytical approaches, and Evidence category for inclusion.

Studies meeting all three criteria—an outdoor or natural setting, structured educational design, and educator or program support—were classified as direct evidence on outdoor education. Other eligible studies examining related outdoor activities, outdoor time, or nature contact were classified as indirect evidence. A summary of the 18 studies is presented in Table 2. Given the heterogeneity in study designs, exposures, participant characteristics, and mental health outcomes, a meta-analysis was not considered appropriate. Therefore, the included studies were synthesized narratively. Consistent with the narrative scope of this mini-review, no formal risk-of-bias assessment tool was applied.

**Table 2:** Empirical studies included for Research Question 2.

**No**.	**References**	Year	Geographical location	Title	Study design	Sample size and age	Data collection method	Data analysis techniques	Findings	Evidence category and rationale
1	Lee et al. (2025)	2025	Hong Kong, China	The effects of a Supporting Wellness in E-Child Learning Environments (SWELE) program on mental wellbeing among children and adolescents with special educational needs and disabilities: a quasi-experimental study	Quantitative research	1,187 youth with special educational needs and disabilities	Psychological and wellbeing scale assessment (Proxy-reports, Pre- and post-tests)	Significance testing and effect size estimation	The Supporting Wellness in E-Child Learning Environments program significantly improved mood, social skills, self-regulation, and playfulness, and reduced anxiety symptoms.	Direct evidence: structured SWELE program and mental wellbeing outcomes.
2	Xu and Jin (2025)	2025	China (secondary students; Beijing Forestry University authorship)	From nature to nurture: how does environmental education link with adolescent problem behaviors through nature connectedness and self-control	Quantitative research	2,361 secondary students (mean age 15.08)	Structured self-report questionnaire survey	Relevant analyses: regression/mediation analysis and moderation effect analysis	Environmental education was negatively associated with problem behaviors; nature connectedness mediated the link, and self-control moderated it.	Indirect evidence: environmental education, nature connectedness, and problem behaviors.
3	Kwok et al. (2021)	2021	Hong Kong, China	Effects of the school-based integrated health promotion program with hydroponic planting on green space use and satisfaction, dietary habits, and mental health in early adolescent students: a feasibility quasi-experiment	Quantitative research	553 secondary school students in Grades 7–9	Questionnaire survey/On-site behavioral observation	Comparison/Regression analysis	Hydroponic planting plus health promotion improved green-space use and competence, life happiness, and some health behavior outcomes.	Direct evidence: school health program with hydroponic planting.
4	Kong and Chen (2024)	2024	Yunnan, China	School garden and instructional interventions foster children's interest in nature	Mixed methods research	24 Grade-4 students in a 4-month school-garden program	On-site behavioral observation/Pre- and post-intervention questionnaire survey	Qualitative and quantitative analyses; thematic analysis	Seventeen of 24 children showed initiated or enhanced interest in nature; school gardens and teacher scaffolding supported curiosity and sustained nature interest.	Direct evidence: school-garden learning with teacher support.
5	Wu et al. (2024a)	2024	Haikou, China	Toward sustainable early education practices: a quasi-experimental study on the effects of kindergarten physical education programs on fundamental movement skills and self-regulation in Haikou City, China	Quantitative research	Kindergarten children from three Haikou kindergartens	Standardized motor skill testing/Psychological scale assessment (Pre- and post-tests)	Differential testing/variance analysis methods	Programs emphasizing structured physical activity improved movement skills and supported self-regulation development.	Direct evidence: structured outdoor physical-activity program with teacher support.
6	Shao et al. (2020)	2020	Shanghai, China	Horticultural activity: its contribution to stress recovery and wellbeing for children	Quantitative research	26 elementary school students (mean age 8.12 years)	Physiological measurements/Psychological state scale assessment	Inter-group difference test	Compared with mobile gaming, horticultural activity reduced physiological stress and increased comfort, relaxation, and wellbeing.	Indirect evidence: horticultural activity, stress recovery, and wellbeing; outdoor setting not clearly established.
7	Guo et al. (2024)	2024	China	Which horticultural activities are more effective for children's recovery from stress and mental fatigue? A quasi-experimental study	Quantitative research	48 elementary students aged 9–12	Physiological measurements/Stress and fatigue self-report scales (Pre- and post-tests)	Comparison of Generalized Estimating Equations (GEE) with between-group/time effects	Horticultural activities produced better stress recovery than the reference task; flower arrangement and sowing/transplanting showed especially robust benefits.	Indirect evidence: horticultural activities and stress recovery; outdoor setting not clearly established.
8	Choe et al. (2025)	2025	Hong Kong, China	Exploring the dose-response relationship between nature-based outdoor activities, nature connectedness and social health in adolescents: a quasi-experimental controlled study	Quantitative research	110 secondary school students aged 13–16	Objective activity duration recording (or wearable tracking)/Structured questionnaire survey	Dose-response comparison between groups	Nature-based interventions yielded an immediate enhancement in nature connectedness; only the 4-day program produced sustained gains and better social connectedness, empathy, and prosocial behavior.	Direct evidence: structured multi-day nature-based outdoor program and social-health outcomes.
9	Li et al. (2026)	2026	Nanjing, China	Mental health profiles and correlates among Chinese adolescents: a latent profile analysis	Quantitative research	9,865 secondary school students	Structured self-report questionnaire survey	Chi-square/Fisher test, multiple logistic regression	Three mental health profiles were identified; outdoor activity time was one of the behaviors distinguishing higher-risk mental health groups.	Indirect evidence: outdoor activity within adolescent mental-health profiles.
10	Wu et al. (2024b)	2024	Haikou, China	The effects of physical exercise on fitness and emotion in Chinese preschoolers	Quantitative research	239 preschoolers (mean age 5.49 years)	On-site physical fitness testing/Emotional state scale assessment	Interactive effect/Variance analysis methods	Structured ball-skills PE and free play improved several fitness outcomes; anxiety-withdrawal outcomes differed by group, with lower anxiety after HS and free-play conditions than ordinary PE.	Direct evidence: structured outdoor physical-activity program with teacher support.
11	Liang et al. (2024)	2024	Southwest China	Association between screen time and physical activity on mental health among preschoolers: a cross-sectional study from Southwest China	Quantitative research	19,015 Chinese preschoolers	Cross-sectional questionnaire survey (Proxy-reported by parents/teachers)s	Logistic regression models	Elevated screen exposure was associated with an increase in adverse mental health outcomes; greater total PA, moderate-to-vigorous physical activity (MVPA), and outdoor activity were generally linked to lower risk, with some gender differences.	Indirect evidence: outdoor activity and preschool mental health.
12	Kong et al. (2026)	2026	China (Grades 5–9 students)	Green exercise and kinesiophobia among Chinese adolescents: the mediating role of self-efficacy	Quantitative research	1,099 Chinese students aged 10–15	Structured questionnaire survey (Including psychological scales)	Descriptive statistics, multiple regression, structural equation modeling (SEM), bias-corrected bootstrap mediation	Increased engagement in nature-based physical activity was correlated with reduced somatic fear and activity avoidance, a relationship partially mediated by enhanced self-efficacy.	Indirect evidence: green exercise, self-efficacy, and kinesiophobia.
13	Zhang et al. (2023)	2023	Shenzhen, China	The link between unhealthy lifestyle behaviors and emotional and behavioral problems in children and adolescents: a latent class analysis	Quantitative research	Children and adolescents in Grades 1–12 from 14 schools in Shenzhen	Cross-sectional questionnaire survey	Latent class analysis and regression analyses	Clusters of unhealthy behaviors were correlated with elevated levels of internalizing and externalizing symptoms; lower physical/outdoor activity was part of riskier lifestyle profiles.	Indirect evidence: low outdoor activity in unhealthy lifestyle profiles.
14	Zhu et al. (2025)	2025	Eastern China (Nantong)	Influencing factors and changing trends of depressive symptoms among middle and junior high school students in Eastern China from 2019 to 2023: a cross-sectional study	Quantitative research	9,920 middle and junior high school students	Large-scale, multiple cross-sectional questionnaire surveys	Single factor/multi-factor analysis, trend chi-square test, and logistic regression	Girls, junior high students, parental absence, < 1 h outdoor activity, and short sleep had higher depressive symptom risk; trends shifted across pandemic years.	Indirect evidence: outdoor activity and depressive symptoms.
15	Wang et al. (2023)	2023	Rural Ningxia, China	The association between screen time and outdoor time on adolescent mental health and academic performance: evidence from rural China	Quantitative research	20,375 rural junior high school students	Questionnaire survey/School academic record extraction	Multivariate ordinary least squares (OLS) regression	Prolonged screen exposure (exceeding 2 h daily) was correlated with deteriorated psychological wellbeing, whereas any duration of outdoor engagement was associated with improved mental states. However, time spent outdoors demonstrated no significant correlation with mathematical achievement.	Indirect evidence: outdoor time and adolescent mental health.
16	Yang et al. (2025)	2025	Yunnan, China	Short sleep duration and daytime outdoor activities effects on adolescents mental health: a stress susceptibility-recovery model analysis	Quantitative research	204,158 adolescents aged 12–18 from 953 schools	Questionnaire survey/Psychological health scale assessment	Multivariable logistic regression, subgroup/interaction tests, and Mendelian randomization	Less daytime outdoor activity and short sleep were linked to worse mental health; short sleep partly explained why lower outdoor activity tracked with emotional problems.	Indirect evidence: daytime outdoor activity and mental health.

## 3 Findings and discussion

### 3.1 Research Question 1: What is outdoor education?

Several studies have defined outdoor education in terms of learning that takes place in outdoor environments. Ford (1986) characterized it as education conducted in, about, and for outdoor settings. Peacock et al. (2021) similarly described outdoor education as educational experiences that occur outdoors and involve interaction with the natural environment. Although the wording varies across studies, the reviewed literature commonly emphasizes the following two features of outdoor education:

1. Outdoor education is primarily an experiential learning pathway occurring in outdoor, natural, or real-world contexts.
2. Outdoor education qualifies as *education* rather than mere outdoor activities due to its typically defined objectives, structured design, teacher or project support, and observable developmental outcomes.

We substantiate these claims by referencing multiple papers from diverse contexts and examining various interpretations.

Regarding the first knowledge proposition, Sharp (1943) emphasized the educational value of direct experiential engagement with nature. Similarly, Burriss and Burriss (2011) defined outdoor education programs as pedagogical initiatives grounded in experiential learning methods, utilizing nature as a learning environment.

Szczepanski (2001) emphasized that learning in outdoor education arises through active participation in authentic environments and through the integration of sensory experience, action, reflection, and prior knowledge. This perspective positions outdoor education as a context-based form of experiential learning, in which children develop understanding by connecting what they observe, hear, and feel with earlier experiences.

Although outdoor education emphasizes experiential learning and openness, it is by no means an arbitrary or unorganized outdoor activity. Ford (1986) believes that the aim of outdoor education is to develop the knowledge, abilities and values of students, so it is inherently growth-oriented. Moreover, in order to distinguish it from general outdoor activities, academic research has proposed the construction of systematic educational programs for outdoor education. As Rickinson et al. (2004) definitively concluded in their seminal review regarding the inherent value of outdoor learning:

Research indicates the value of programmes which: (i) provide longer, more sustained outdoor experiences than is often provided; (ii) incorporate well-designed preparatory and follow-up work; (iii) use a range of carefully-structured learning activities and assessments linked to the school curriculum; (iv) recognize the crucial role of the teacher/educator. (p. 8)

Rickinson et al. (2004) believe that activities are not necessarily outdoor education simply because they take place outdoors. On the other hand, these activities are also given clear learning goals, organized in a certain manner, and focused on achieving specific outcomes, thus meeting the conditions of educational outdoor learning.

The intervention reported by Kwok et al. (2021) increased life happiness but did not significantly reduce depressive symptoms or anxiety. A school-based all-round program was conducted that integrated hydroponic cultivation into health education and was not treated as an independent project. This intervention system had a clear implementation cycle, a project schedule and evaluation indicators. Therefore, the actual educational value of the program does not come from the spatial features of the outdoor area but rather from its specific development goals, well-arranged design, and achievable, results-oriented mechanisms.

In summary, looking at the relevant literature, it can be seen that the concept of *outdoor education* generally includes elements such as nature, organization, and experience. That is to say, to help students gain knowledge through direct exposure to the environment, active participation in activities, multi-sensory stimulation, reflection, etc. Secondly, because it generally has clear goals, an organized structure, teacher or project support, and focuses on observable changes in children's development, outdoor education is considered *education* and not just an outdoor activity.

Of course, reaching a broad consensus on the essence of outdoor education remains challenging due to the numerous conceptual variations in its definition. However, if authors could elaborate by incorporating common theoretical frameworks within the definition of outdoor education, the entire theoretical system would become more coherent, clear, and consistent. The next section draws on direct evidence together with indirect evidence to explore the potential mental health implications of outdoor education for Chinese children.

### 3.2 Research Question 2: What potential impacts may outdoor education have on the mental health outcomes of children in China?

Having clarified the concept of outdoor education, we now turn to its possible impacts upon the psychological wellness of Chinese children. Becker et al. (2017) and Mygind et al. (2018) both suggest that outdoor education programs appear to have multiple positive effects on students' identity formation, mental health status, stress relief, social skills. Direct evidence is limited. Most of the available studies provide indirect supporting evidence from nature exposure, horticultural activity, outdoor physical activity, or other related outdoor experiences. Based on the reviewed evidence, we propose the following two interpretations:

1. Outdoor education may support positive psychological outcomes through continuous nature exposure, outdoor physical activity, and social interaction.
2. The mental health effects of outdoor education may be primarily manifested in enhanced positive emotions, improved social connections, greater self-regulation, and improved overall psychological wellbeing.

Subsequently, each of these assertions is substantiated with evidence drawn from the synthesized literature. We begin with the first perspective. Choe et al. (2025) linked a structured nature-based outdoor program with greater nature connectedness and social health agement, as well as ecological awareness and perspectives. Furthermore, dose-response studies have found that longer periods of nature-based outdoor activity can strengthen adolescents' sense of nature connectedness and further improve social health (Choe et al., 2025). This direct evidence suggests that the potential mental health relevance of outdoor education may depend less on brief exposure alone and more on whether outdoor experiences are organized, sustained, and repeated.

However, we do not deny that short-term natural or horticultural activities can also lead to immediate stress recovery and positive emotional changes. For instance, Shao et al. (2020) provided indirect evidence that even a 5-min plant-related task could enhance children's sense of relaxation and reduce perceived stress levels.

It is essential to acknowledge that outdoor learning does not spontaneously foster emotional health. Putting children in a natural environment by themselves is not enough either. In fact, the included studies have shown that outdoor education can gradually create a path for psychological enhancement by strengthening natural connections, social bonds, peer interactions, and teacher-led exploratory experiences (Kong and Chen, 2024; Lee et al., 2025; Xu and Jin, 2025). This process involves multiple mechanisms working synergistically rather than relying on a single factor. As Mann et al. (2022) emphasized:

“various manifestations of NSLOtC can effectively produce holistic learning, well-being and development outcomes.” (p. 9)

Meanwhile, additional studies suggest that this pathway has a more nuanced dimension. Recovery experiences, sleep improvement, self-efficacy, self-control, and self-regulation are likely all critical components (Kong et al., 2026; Yang et al., 2025; Wu et al., 2024a,b). These supplementary studies alone cannot establish the efficacy of outdoor education interventions. Although these are not direct examples of outdoor education, they suggest that the impact of outdoor education on mental health may result from the combined effects of multiple factors.

To elucidate the impact of outdoor education on the mental health of Chinese children, it is essential to consider not only the mechanisms of action but also the outcomes. The literature provides preliminary evidence for understanding the possible mental health benefits of outdoor education and related outdoor activities among Chinese children. Horticultural activities closely related to outdoor learning have been associated with relaxation and reduced stress (Shao et al., 2020). A four-day nature-based outdoor activity program was associated with social connectedness, empathy, and prosocial behavior (Choe et al., 2025). Thus, structured nature-based outdoor experiences may support social connectedness and prosocial behavior, but evidence for emotional development remains limited. Based on this, some scholars believe that the primary objective of outdoor education is to improve children happiness and thus promote their mental health development at a general level. However, research results are mixed. For example, Kwok et al. (2021) found that although the intervention increased students' life happiness, it did not significantly improve predefined clinical indices of mental health, such as depressive symptoms and anxiety disorders.

In summary, outdoor education is a structured practice that combines natural settings, organized learning, and developmental support, rather than simply moving activities outdoors. Its mental-health relevance may depend on sustained engagement, social interaction, educator support, and developmental processes. Because many included studies examined related nature exposure or outdoor activities rather than outdoor education itself, their findings constitute indirect evidence and should be interpreted cautiously. Implementation should also address accessibility and safety, particularly for children with chronic diseases or special needs, through individualized preparation, appropriate supervision, and emergency planning (Daniş and Kudu, 2023). This consideration concerns implementation and generalizability, not evidence of mental-health efficacy.

## 4 Conclusion and limitation

This review clarifies the definition of outdoor education and examines its potential impact on Chinese children's mental health. Distinguishing outdoor education from related outdoor and nature-based activities addresses conceptual ambiguity that could cause misinterpretation in future research and practice.

Available evidence suggests that structured outdoor and nature-based educational activities may be associated with favorable emotional, social, and self-regulatory outcomes in Chinese children. However, confidence is limited by heterogeneous study designs and outcome measures, predominantly observational or indirect evidence, and limited long-term follow-up. Searching only two English-language databases may have excluded relevant studies, and no formal risk-of-bias assessment was conducted. These limitations warrant cautious interpretation.

Future research should move beyond broad associations between outdoor exposure and mental health to evaluate clearly defined outdoor education interventions in Chinese schools. Studies should specify educational objectives, learning activities, educator support, natural settings, intervention frequency, and duration. Becker et al. (2017) recommended larger quasi-experimental, randomized controlled, and longitudinal studies of school- and curriculum-based outdoor education. Similarly, Mann et al. (2022) called for rigorous research distinguishing nature-specific outdoor learning from classroom settings and identifying environmental and pedagogical elements contributing to outcomes. Accordingly, prospective studies in China should use appropriate comparison groups, validated mental health measures, and longer follow-up periods. They should also examine how outcomes vary by age, school context, and program design.

## References

[B1] Barfod K. S. Bolling M. Mygind L. Elsborg P. Ejbye-Ernst N. Bentsen P. (2021). Reaping fruits of labour: revisiting education outside the classroom provision in Denmark upon policy and research interventions. Urban For. Urban Green. 60:127044. doi: 10.1016/j.ufug.2021.127044

[B2] Becker C. Lauterbach G. Spengler S. Dettweiler U. Mess F. (2017). Effects of regular classes in outdoor education settings: a systematic review on students' learning, social and health dimensions. Int. J. Environ. Res. Public Health 14:485. doi: 10.3390/ijerph14050485 28475167 PMC5451936

[B3] Bentsen P. Mygind E. Randrup T. B. (2009). Towards an understanding of udeskole: education outside the classroom in a danish context. Education 37, 29–44. doi: 10.1080/03004270802291780

[B4] Burriss K. G. Burriss L. L. (2011). Outdoor play and learning: policy and practice. Int. J. Educ. Policy Leadersh. 6, 1–13. doi: 10.22230/ijepl.2011v6n8a306

[B5] Choe E. Y. Lee J. Y. Zhu S. (2025). Exploring the dose-response relationship between nature-based outdoor activities, nature connectedness and social health in adolescents: a quasi-experimental controlled study. J. Adolesc. 97, 1518–1529. doi: 10.1002/jad.12515 40365936 PMC12318473

[B6] Daniş F. Kudu E. (2023). Nature activities with special child groups. Anatol. J. Emerg. Med. 6, 92–97. doi: 10.54996/anatolianjem.1300695

[B7] Down M. J. Picknoll D. Edwards T. Farringdon F. Hoyne G. Piggott B. . (2025). Outdoor adventure education for adolescent social and emotional wellbeing: a systematic review and meta-analysis. J. Adventure Educ. Outdoor Learn. 25, 869–898. doi: 10.1080/14729679.2024.2386350

[B8] Fellmeth G. Rose-Clarke K. Zhao C. Busert L. K. Zheng Y. Massazza A. . (2018). Health impacts of parental migration on left-behind children and adolescents: a systematic review and meta-analysis. Lancet 392, 2567–2582. doi: 10.1016/S0140-6736(18)32558-3 30528471 PMC6294734

[B9] Ford P. M. (1986). Outdoor education: definition and philosophy. ERIC Clearinghouse on Rural Education and Small Schools. Available online at: https://eric.ed.gov/?id=ED267941 (Accessed September 7, 2026).

[B10] Galderisi S. Heinz A. Kastrup M. Beezhold J. Sartorius N. (2015). Toward a new definition of mental health. World Psychiatry 14, 231–233. doi: 10.1002/wps.20231 26043341 PMC4471980

[B11] Guo L. Xu W. Shi Y. Gao S. Xiao C. Zhang X. . (2024). Which horticultural activities are more effective for children's recovery from stress and mental fatigue? A quasi-experimental study. Front. Psychol. 15:1352186. doi: 10.3389/fpsyg.2024.1352186 38680274 PMC11050040

[B12] Hao L. Ludin A. F. M. Ahmad M. Meng X. Lei H. Z. (2024). The prevalence and its associated factors of psychological stress among middle school students in China: pooled evidence from a systematic scoping review. Front. Public Health 12:1358210. doi: 10.3389/fpubh.2024.1358210 38694991 PMC11062323

[B13] Hu R. Mou S. (2025). Outdoor education for sustainable development: a systematic literature review. Sustainability 17:3338. doi: 10.3390/su17083338

[B14] Kong C. Chen J. (2024). School garden and instructional interventions foster children's interest in nature. People Nat. 6, 712–732. doi: 10.1002/pan3.10597

[B15] Kong M. Ren Y. Liu T. Yin Z. (2026). Green exercise and kinesiophobia among chinese adolescents: the mediating role of self-efficacy. Front. Psychol. 17:1743182. doi: 10.3389/fpsyg.2026.1743182 41685214 PMC12891208

[B16] Kwok S. W. H. Wu C. S. C. Tong H. T. Ho C. N. Leung K. L. Leung Y. C. P. . (2021). Effects of the school-based integrated health promotion program with hydroponic planting on green space use and satisfaction, dietary habits, and mental health in early adolescent students: a feasibility quasi-experiment. Front. Public Health 9:740102. doi: 10.3389/fpubh.2021.740102 34631651 PMC8498580

[B17] Lee R. L. T. Leung A. W. Y. Chong Y. Y. Chan S. W. C. Choi K. C. Chien W. T. (2025). The effects of a SWELE program on mental wellbeing among children and adolescents with special educational needs and disabilities: a quasi-experimental study. J. Appl. Res. Intellect. Disabil. 38:e70164. doi: 10.1111/jar.70164 41414736 PMC12715380

[B18] Li F. Cui Y. Li Y. Guo L. Ke X. Liu J. . (2022). Prevalence of mental disorders in school children and adolescents in China: diagnostic data from detailed clinical assessments of 17,524 individuals. J. Child Psychol. Psychiatry 63, 34–46. doi: 10.1111/jcpp.13445 34019305

[B19] Li X. Li J. Ye S. Liu H. Yang Y. Lu X. . (2026). Mental health profiles and correlates among Chinese adolescents: a latent profile analysis. Public Health 251:106121. doi: 10.1016/j.puhe.2025.106121 41468735

[B20] Liang Y. Jin Q. Miao J. Ni X. Qian X. Xiong Y. . (2024). Association between screen time and physical activity on mental health among preschoolers: a cross-sectional study from southwest China. BMC Public Health 24:261. doi: 10.1186/s12889-024-17722-8 38254090 PMC10804503

[B21] Mann J. Gray T. Truong S. Brymer E. Passy R. Ho S. . (2022). Getting out of the classroom and into nature: a systematic review of nature-specific outdoor learning on school children's learning and development. Front. Public Health 10:877058. doi: 10.3389/fpubh.2022.877058 35651851 PMC9149177

[B22] Ministry of Education, National Development and Reform Commission, Ministry of Public Security, Ministry of Finance, Ministry of Transport, Ministry of Culture, et al. (2016). Opinions on promoting study tours for primary and secondary school students. Available online at: https://hudong.moe.gov.cn/srcsite/A06/s3325/201612/t20161219_292354.html (Accessed September 7, 2026).

[B23] Mygind L. Stevenson M. P. Liebst L. S. Konvalinka I. Bentsen P. (2018). Stress response and cognitive performance modulation in classroom versus natural environments: a quasi-experimental pilot study with children. Int. J. Environ. Res. Public Health 15:1098. doi: 10.3390/ijerph15061098 29843433 PMC6025376

[B24] Page M. J. McKenzie J. E. Bossuyt P. M. Boutron I. Hoffmann T. C. Mulrow C. D. . (2021). The PRISMA 2020 statement: an updated guideline for reporting systematic reviews. BMJ 372:n71. doi: 10.1136/bmj.n71 33782057 PMC8005924

[B25] Peacock J. Bowling A. Finn K. McInnis K. (2021). Use of outdoor education to increase physical activity and science learning among low-income children from urban schools. Am. J. Health Educ. 52, 92–100. doi: 10.1080/19325037.2021.1877222

[B26] Priest S. (1986). Redefining outdoor education: a matter of many relationships. J. Environ. Educ. 17, 13–15. doi: 10.1080/00958964.1986.9941413

[B27] Rao W.-W. Xu D.-D. Cao X.-L. Wen S.-Y. Che W.-I. Ng C. H. . (2019). Prevalence of depressive symptoms in children and adolescents in China: a meta-analysis of observational studies. Psychiatry Res. 272, 790–796. doi: 10.1016/j.psychres.2018.12.133 30832200

[B28] Rickinson M. Dillon J. Teamey K. Morris M. Choi M. Y. Sanders D. . (2004). A Review of Research on Outdoor Learning. Shrewsbury: National Foundation for Educational Research and King's College London.

[B29] Samsudin S. Kamalden T. Aziz A. Ismail H. Ujang A. (2019). Effectiveness of outdoor education program on physical education student resilience. Int. J. Innov. Creat. Change 9, 218–229.

[B31] Sharp L. B. (1943). Outside the classroom. Educ. Forum 7, 361–368. doi: 10.1080/00131724309340840

[B30] Shao Y. Elsadek M. Liu B. (2020). Horticultural activity: its contribution to stress recovery and wellbeing for children. Int. J. Environ. Res. Public Health 17:1229. doi: 10.3390/ijerph17041229 32074999 PMC7068549

[B32] Szczepanski A. (2001). “What is outdoor education?,” in Other Ways of Learning: Outdoor Adventure Education and Experiential Learning in School and Youth Work: Proceedings of the 4th European Congress for Outdoor Adventure Education and Experiential Learning (Rimforsa: European Institute for Outdoor Adventure Education and Experiential Learning), 15–18.

[B33] Tang Y.-Y. Hölzel B. K. Posner M. I. (2015). The neuroscience of mindfulness meditation. Nat. Rev. Neurosci. 16, 213–225. doi: 10.1038/nrn3916 25783612

[B34] UNICEF (1989). Convention on the rights of the child. Available online at: https://www.ohchr.org/en/instruments-mechanisms/instruments/convention-rights-child (Accessed September 7, 2026).

[B35] Wang H. Abbey C. Kennedy T. Feng E. Li R. Liu F. . (2023). The association between screen time and outdoor time on adolescent mental health and academic performance: evidence from rural china. Risk Manag. Healthc. Policy 16, 369–381. doi: 10.2147/RMHP.S384997 36923495 PMC10010124

[B36] Wu H. Eungpinichpong W. Ruan H. Chen W. Yang Y. Dong X. (2024a). Towards sustainable early education practices: a quasi-experimental study on the effects of kindergarten physical education programs on fundamental movement skills and self-regulation in Haikou City, China. Sustainability 16:1400. doi: 10.3390/su16041400

[B37] Wu H. Ruan H. Eungpinichpong W. Zhou W. (2024b). The effects of physical exercise on fitness and emotion in Chinese preschoolers. BMC Public Health 24:2407. doi: 10.1186/s12889-024-19895-8 39232695 PMC11375933

[B38] Xu W. Jin C. (2025). From nature to nurture: how does environmental education link with adolescent problem behaviors through nature connectedness and self-control. Youth Soc. 57, 784–808. doi: 10.1177/0044118X251392252

[B39] Yang Z. Whatman S. (2025). Development and validation of standards for evaluating the quality of qualitative research on Olympics breakdance. Humanit. Soc. Sci. Commun. 12:471. doi: 10.1057/s41599-025-04792-1

[B40] Yang X. Yang Y. Yang J. Ni J. Li H. Mu X. . (2025). Short sleep duration and daytime outdoor activities effects on adolescents mental health: a stress susceptibility-recovery model analysis. J. Affect. Disord. 382, 428–437. doi: 10.1016/j.jad.2025.04.085 40274127

[B41] Zhang Y. Wang Q. Zhu Z. Zong Q. Wu X. Tao F. (2023). The link between unhealthy lifestyle behaviors and emotional and behavioral problems in children and adolescents: a latent class analysis. Psychol. Health Med. 28, 2182–2197. doi: 10.1080/13548506.2023.2229979 37394763

[B42] Zhu X. An N. Tang Z. Huang J. Ren Q. Wu Y. (2025). Influencing factors and changing trends of depressive symptoms among middle and junior high school students in eastern China from 2019 to 2023: a cross-sectional study. BMC Public Health 25:17. doi: 10.1186/s12889-024-21252-8 39748301 PMC11697880

